# Isolated partial anomalous pulmonary venous return with severe mitral stenosis managed successfully by a modified Warden procedure and mitral valve replacement: a rare case report

**DOI:** 10.1097/RC9.0000000000001093

**Published:** 2026-09-29

**Authors:** Syeda A. Rizvi, Sundia Siddique, Hina Aslam, Muhammad H. Javaid, Hasibullah Aminpoor, Hasiba Karimi

**Affiliations:** a Department of Surgery, Karachi Medical and Dental College, Karachi, Pakistan; b Department of Surgery, Shifa College of Medicine, Islamabad, Pakistan; c Faculty of Medicine, Kabul University of Medical Sciences Abu Ali Ibn Sina, Kabul, Afghanistan; d Faculty of Medicine, Bezmialem Vakif University, Istanbul, Turkey

**Keywords:** mitral stenosis, mitral valve replacement (MVR), modified Warden procedure, partial anomalous pulmonary venous return (PAPVR), rheumatic heart disease (RHD), superior vena cava (SVC)

## Abstract

**Background:**

Partial anomalous pulmonary venous return (PAPVR) is a very rare condition, with an incidence ranging from 0.4% to 0.7% in autopsy series. Isolated PAPVR without autism spectrum disorder is extremely rare, accounting for less than 10% of all PAPVR.

**Case presentation:**

The patient was a 40-year-old female with isolated PAPVR, rheumatic heart disease, and hypertension who presented with New York Heart Association (NYHA) Class IV symptoms 15 years after failed percutaneous transvenous mitral commissurotomy. Multimodality imaging showed anomalous drainage of the right superior pulmonary vein into the superior vena cava (SVC) with aneurysmal dilation (65 × 35 mm), a marked left-to-right shunt (pulmonary blood flow to systemic blood flow ratio of 7.25), and severe mitral stenosis (mitral valve area 0.8 cm^2^; a Wilkins score of 9/16). She underwent a modified Warden procedure and mechanical mitral valve replacement using a 27 mm bileaflet valve. The postoperative course was uneventful. After 12 months, she improved to NYHA Class I, with a prosthetic mean gradient of 4 mmHg, a normal right ventricular size, and a low systolic pulmonary artery pressure (38 mmHg). No arrhythmias, SVC obstruction, or prosthetic dysfunction were noted.

**Conclusion:**

This is an example of the complicated hemodynamic relationship between PAPVR and severe rheumatic mitral stenosis. The two conditions can be repaired together successfully with careful multimodality imaging assessment and an individualized surgical plan.

## Introduction

Partial anomalous pulmonary venous return (PAPVR) is a congenital malformation of the heart characterized by drainage of the pulmonary veins into systemic veins rather than into the left atrium. This condition occurs in about 0.4%–0.7% of autopsies^[^1^]^. About 90% of cases have an autism spectrum disorder (ASD), typically the sinus venosus form^[^2,3^]^. An isolated case of PAPVR that does not involve communication between the atria is rare (<10% of cases)^[^3^]^.

HIGHLIGHTS

- Isolated partial anomalous pulmonary venous return (PAPVR) without an atrial septal defect is exceedingly rare in adults.
- Concomitant severe rheumatic mitral stenosis with PAPVR creates a complex left- and right-sided hemodynamic burden.
- A markedly elevated pulmonary blood flow to systemic blood flow ratio signifies a significant left-to-right shunt requiring definitive surgical correction.
- The combined modified Warden procedure and mitral valve replacement provide effective and durable clinical outcomes.

In contrast to ASD-related PAPVR, isolated PAPVR lacks a decompressive connection between the atria, resulting in continuous left-to-right shunting via the right side of the heart. Right ventricular hypertrophy occurs because of excessive volume loading of the right side, with onset typically between ages 30 and 50 years^[^4,5^]^.

Treatment for the supracardiac form, where anomalous pulmonary veins drain through the superior vena cava (SVC), usually involves performing either the Warden procedure or one of its variations. The surgical treatment method allows the transfer of pulmonary venous blood to the left atrium while simultaneously anastomosing the distal SVC to the right atrial appendage to avoid sinoatrial node damage^[^6^]^. Surgery is warranted for symptomatic cases or patients with a pulmonary blood flow to systemic blood flow ratio (Qp:Qs) of more than 1.5:1 with right ventricular enlargement^[^7^]^.

In both PAPVR and sinus venosus ASD, the congenital basis arises from abnormal incorporation of the pulmonary veins into the sinus venosus^[^2^]^. The coexistence of isolated PAPVR and rheumatic mitral stenosis is extremely rare. In our case study, we present a 40-year-old woman with isolated PAPVR draining into the SVC and severe recurrent rheumatic mitral stenosis post-percutaneous transvenous mitral commissurotomy (PTMC), managed with a modified Warden procedure and mechanical mitral valve replacement (MVR) in accordance with the Surgical CAse REport 2025 criteria^[^8^]^.

## Case presentation

A case report involves a 40-year-old female patient with documented PAPVR without ASD, rheumatic heart disease, and hypertension, who was admitted with progressive dyspnea, orthopnea, and chest pain for 2 months. She had undergone PTMC for rheumatic mitral stenosis in the past and had initial improvement, but it progressively worsened over 2 years to develop dyspnea at rest.

She had New York Heart Association (NYHA) class IV on presentation, with BP 130/80 mmHg, HR 92/min, RR 22/min, and oxygen saturation of 91% on room air. On examination, there was elevated jugular venous pressure, pitting edema up to the mid-shins, and a right ventricular impulse. The cardiac examination revealed a loud S1, an opening snap, and a mid-diastolic murmur with presystolic accentuation, findings suggestive of severe mitral stenosis. Basal crackles were present bilaterally.

### Diagnostic assessment

Possible differential diagnoses included: (1) rheumatic mitral stenosis with severe, decompensated right ventricular failure; (2) PAPVR with chronic right-sided volume overload; (3) both combined (the patient had both conditions); (4) pulmonary hypertension with postcapillary and precapillary components; and (5) cor triatriatum or a left atrial tumor with functional occlusion, which was excluded on imaging.

### Investigations

Transthoracic echocardiography revealed a left ventricular ejection fraction (LVEF) of 55%, a hyperdynamic right ventricle [tricuspid annular plane systolic excursion (TAPSE 30 mm)], severe left atrial enlargement (86 mL/m^2^), and dilation of the right-sided cardiac chambers. No interatrial septal defect was found. Mitral valve stenosis was severe (valve area 0.8 cm^2^, gradient 18 mmHg, Wilkins score 9/16). Trace regurgitation was also present. The estimated pulmonary artery systolic pressure (PASP) was 65 mmHg.

Transesophageal echocardiography (TEE) documented that the patient had significant mitral stenosis and aberrant drainage of the dilated right upper pulmonary vein into the SVC, with a constant left-to-right shunt and no ASD. The right heart catheterization showed the following results: Qp:Qs ratio = 7.25, pulmonary artery pressure = 42 mmHg, pulmonary capillary wedge pressure (PCWP) = 28 mmHg, and PVR = 3.2 Wood units, indicating reactive pulmonary hypertension.

A 65 × 35 mm right upper lobe pulmonary vein aneurysm with drainage to the lateral SVC and no drainage to the left atrium was found on contrast-enhanced (CE) computed tomography (CT). The Electrocardiogram revealed sinus rhythm with right ventricle hypertrophy and right axis deviation. There were no abnormal findings on laboratory testing.

### Management

After multidisciplinary discussion, a decision for surgical intervention was made based on the 2018 AHA/ACC recommendations for Adult Congenital Heart Disease. The combination of a Modified Warden repair and mechanical MVR was chosen because (1) the patient had severe mitral regurgitation that was not amenable to repeat PTMC; (2) the Qp:Qs ratio was > 7; and (3) the patient’s age favored mechanical valve placement.

The surgery was performed using a median sternotomy with cardiopulmonary bypass (195 minutes of cardiopulmonary bypass, 138 minutes of cross-clamp time, and hypothermia at 32°C). Del Nido cardioplegia was administered.

Intraoperative findings revealed that the only abnormality was PAPVR, with the right upper pulmonary vein draining into the SVC. The SVC was cut above the anomalous insertion, and the flow was directed into the left atrium by creating a pericardial baffle through an ASD. The distal SVC was anastomosed to the right atrial appendage.

The diseased, calcified valve was replaced with a 27 mm mechanical valve prosthesis. Post-procedure TEE was consistent with normal function (gradient of 4 mmHg) and no leakage. Weaning from bypass occurred under low-dose inotropic coverage for 24 hours.

### Follow-up and outcomes

Recovery after surgery was uncomplicated. The tracheostomy was removed on day 1, and the patient was transferred to step-down care on day 3. Heparin therapy began on day 1, and Coumadin was initiated on day 2 with an international normalized ratio (INR) target of 2.5–3.5, together with 100 mg of aspirin per day. A therapeutic INR was achieved before discharge on day 10.

At 12 months post-procedure, the patient was asymptomatic (NYHA Class I). The echocardiographic assessment indicated an LVEF of 62%, a decrease in SPAP from 65 to 38 mmHg, normalization of right ventricular measurements, a TAPSE of 22 mm, and a functioning mitral prosthesis (mean gradient 4 mmHg). left atrial volume index (LAVI) decreased from 86 to 58 mL/m^2^. No arrhythmias, SVC obstruction, or prosthesis-related problems were observed. CE chest CT demonstrated an aneurysmal right upper pulmonary vein measuring 65 × 35 mm, draining directly into the SVC, with no connection to the left atrium (Fig. 1).

**Figure 1.**
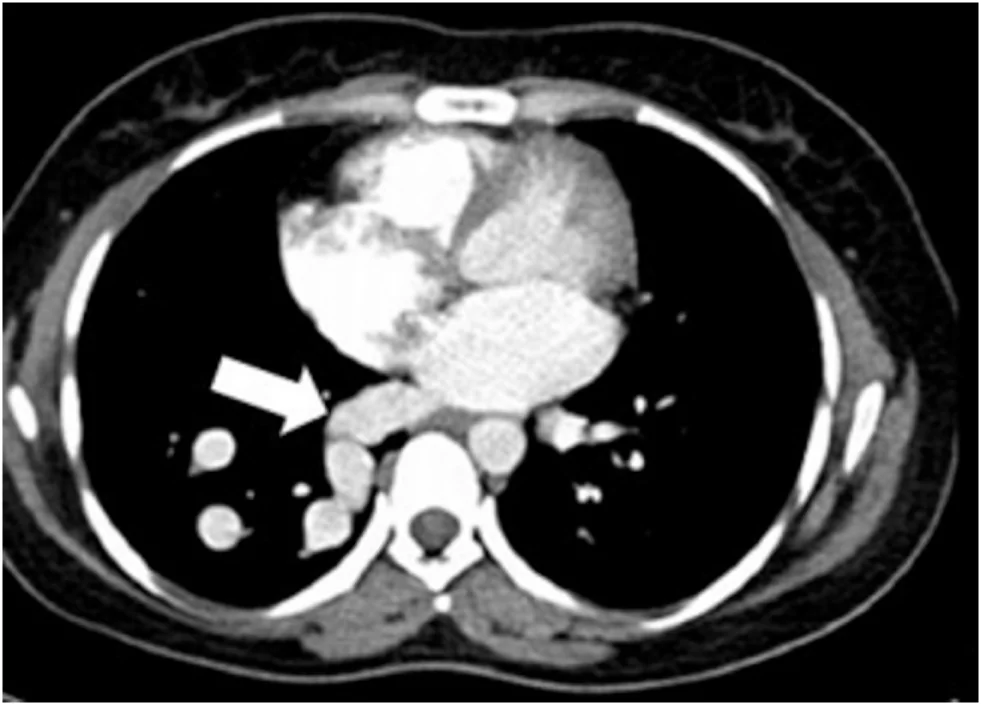
CT showing an aneurysmal right upper pulmonary vein (65 × 35 mm) draining into the SVC (arrow), with no left atrial connection. CT, computed tomography. SVC, superior vena cava.

## Discussion

This is a unique case of isolated PAPVR to the SVC without any ASD, along with severe recurrent rheumatic mitral stenosis, leading to volume and pressure overload of both ventricles, accompanied by NYHA class IV symptoms and pulmonary hypertension.

Unlike ASD-associated PAPVR, isolated PAPVR does not have any decompression between the atria, leading to constant pressure on the right side of the heart, causing remodeling of the pulmonary vessels^[^4,5^]^. An extremely high Qp:Qs ratio of 7.25 in the patient indicates a large shunt volume, possibly due to aneurysmal dilatation of the anomalous vein. A comparison with the literature is provided below in Table 1.

**Table 1:** Summary of reported cases of PAPVR without ASD and associated mitral/valvular pathology.

Author/year	Age/sex	ASD	Anomalous vein site	Qp:Qs	Associated pathology	Procedure	Outcome at follow-up
Urushadze *et al*, 2015^[^1^]^	NR	No	Right pulmonary veins	NR	None specified	Surgical correction	Favorable (NR)
Broy & Bennett, 2008^[^5^]^	Adult/M	NR	RSPV → SVC	NR	Exertional dyspnea	Surgical repair	Symptom resolution
Sears *et al*, 2012^[^9^]^	43/F	No	RSPV → SVC	2.8	Adult-onset PAH	Warden procedure	Improved; PAP normalized
Ebrahimi *et al*, 2025^[^10^]^	Adult/NR	No	Anomalous LSPV	NR	Incidental/atypical	Surgical correction	Favorable at 6 months
Present Case, 2025	40/F	No	RSPV → SVC (aneurysm 65 × 35 mm)	7.25	Severe rheumatic MS, prior PTMC, PAH (SPAP 65 mmHg)	Modified Warden + MVR (27 mm mechanical)	NYHA I; SPAP 38 mmHg; mean gradient 4 mmHg at 12 months

ASD, atrial septal defect; LSPV, left superior pulmonary vein; MS, mitral stenosis; MVR, mitral valve replacement; NR, not reported; PAH, pulmonary arterial hypertension; PAPVR, partial anomalous pulmonary venous return; PTMC, percutaneous transvenous mitral commissurotomy; RSPV, right superior pulmonary vein; SPAP, systolic pulmonary artery pressure; SVC, superior vena cava.

The rheumatic mitral stenosis also contributed to higher pressure in the left atrium. Therefore, there were two components of the pulmonary hypertension: pre-capillary and post-capillary. The combination of both led to severe hemodynamic dysfunction (PASP, 65 mmHg; mean pulmonary artery pressure, 42 mmHg; PCWP, 28 mmHg). Such coexistence is rarely reported^[^2,3^]^.

Diagnosis required multimodal imaging. TTE identified structural abnormalities and pulmonary hypertension; TEE confirmed anomalous drainage and excluded an ASD; CT defined the anatomy for surgery; and catheterization quantified shunt severity and vascular resistance. Magnetic resonance imaging was deferred owing to sufficient diagnostic clarity.

With a history of PTMC, a high Wilkins score, and a substantial shunt burden, a combined approach using modified Warden’s repair and MVR would have been the most appropriate management option according to existing guidelines^[^6,7,9^]^.

## Limitations

A limitation of this case report is that it is an uncontrolled single-case study. A follow-up period of 12 months is inadequate to evaluate the long-term durability of the prosthesis or to assess pulmonary vascular remodeling. The absence of cardiac magnetic resonance imaging precluded accurate estimation of right ventricular volumes.

## Conclusion

In this report, there is a unique association of isolated PAPVR without ASD, along with severe rheumatic mitral stenosis with recurrent episodes, leading to severe dysfunction of both ventricles and severe pulmonary hypertension. In our patient, the combined procedures of the modified Warden technique for PAPVR and mechanical MVR achieved very good results; she is now NYHA Class I with normal right ventricular size and a reduction in pulmonary arterial pressure (from 65 to 38 mmHg), and stable prosthetic function at 1-year follow-up.

## Data Availability

Data available on request from authors.

## Patient’s perspective

The patient reported a profound and life-changing improvement in her quality of life and functional capacity following the procedure. She described complete elimination of the dyspnea and peripheral oedema that had previously rendered her dependent and housebound and expressed that her current functional capacity was equivalent to her premorbid baseline.
